# Internal Use of Chloroform in Convulsions

**Published:** 1866-01

**Authors:** 


### Internal use of Chloroform in Convulsions.

The *American Medical Journal* for October contains an interesting paper by Dr. A. P. Merrill of New York, on the internal use of chloroform, in convulsions dependent on cerebral congestion. He gives it in large doses, often one and even two drachms at a dose. When thus administered he ascribes to it a decided power over every kind of convulsive moment, and certain relief to every form of congestion. It is much less irritating to the mucous lining of the mouth and throat than to the skin. "Sometimes a single drop falling into the folds of the neck will cause vesication, while a fluid drachm passing into the stomach gives only a slight inconvenience by its stimulation of the mouth and throat." In a case of convulsion occurring in a little girl, and which had continued two hours, the child being pulseless and apparently at the point of death, he "poured full half a drachm within her lips, which were elevated to receive it. It found its way slowly through the teeth, and was, with a convulsive effort, swallowed without any loss. In one minute all the convulsive movements were lessened, as remarked by the attendants. Still, there remained considerable spasmodic action, and the eyes were unaffected, being wide open, with dilated pupils. The dose of chloroform was repeated in a few minutes, and almost instantly her eyes were closed and no spasm remained." Other cases are reported of like character. The remedy was equally efficacious in congestive chill, and it was used with success in two cases of poisoning by strichnia. We confess to some degree of surprise at this heroic mode of administering chloroform. We have always been cautious to dilute it liberally with mucilage. But the statements of Dr. Merrill certainly entitle his method to a full trial at the hands of others. Though the convulsions of children are rarely fatal, yet we do occasionally meet with an attack which resists all treatment and holds on tenaciously till death. If the large doses of chloroform can be applied in such cases with the declared results, we shall rejoice to have in our possession the means of achieving such a triumph.—*Pacific Medical Journal.*

